# On-chip large-scale all-optical interconnect for ultra-low-latency deep neural network inference

**DOI:** 10.1093/nsr/nwag282

**Published:** 2026-05-19

**Authors:** Zihan Tao, Yan Zhou, Weizhen Yu, Huajin Chang, Hao Wu, Ailian Cheng, Qian Hong, Yonglin Tang, Xiaolin Bian, Lingwei Meng, Le Li, Gaoang Shen, Cheng Zhang, Yongguang Huang, Wenhua Wang, Haowen Shu, Xingjun Wang

**Affiliations:** State Key Laboratory of Photonics and Communications, School of Electronics, Peking University, Beijing 100871, China; Key Laboratory for Advanced Optoelectronic Integrated Chips of Jiangsu Province, Peking University Yangtze Delta Institute of Optoelectronics, Nantong 226010, China; State Key Laboratory of Photonics and Communications, School of Electronics, Peking University, Beijing 100871, China; State Key Laboratory of Photonics and Communications, School of Electronics, Peking University, Beijing 100871, China; Key Laboratory for Advanced Optoelectronic Integrated Chips of Jiangsu Province, Peking University Yangtze Delta Institute of Optoelectronics, Nantong 226010, China; Hangzhou Flyslice Technologies Co., Ltd, Hangzhou 330113, China; Henan Key Laboratory of Optoelectronic Circuit and Integration, Henan Shijia Photons Technology Co., Ltd, Hebi 458000, China; Hangzhou Flyslice Technologies Co., Ltd, Hangzhou 330113, China; State Key Laboratory of Photonics and Communications, School of Electronics, Peking University, Beijing 100871, China; Key Laboratory for Advanced Optoelectronic Integrated Chips of Jiangsu Province, Peking University Yangtze Delta Institute of Optoelectronics, Nantong 226010, China; State Key Laboratory of Photonics and Communications, School of Electronics, Peking University, Beijing 100871, China; State Key Laboratory of Photonics and Communications, School of Electronics, Peking University, Beijing 100871, China; State Key Laboratory of Photonics and Communications, School of Electronics, Peking University, Beijing 100871, China; Henan Key Laboratory of Optoelectronic Circuit and Integration, Henan Shijia Photons Technology Co., Ltd, Hebi 458000, China; Hangzhou Flyslice Technologies Co., Ltd, Hangzhou 330113, China; State Key Laboratory of Photonics and Communications, School of Electronics, Peking University, Beijing 100871, China; Frontiers Science Center for Nano-optoelectronics, Peking University, Beijing 100871, China; State Key Laboratory of Photonics and Communications, School of Electronics, Peking University, Beijing 100871, China; Key Laboratory for Advanced Optoelectronic Integrated Chips of Jiangsu Province, Peking University Yangtze Delta Institute of Optoelectronics, Nantong 226010, China; Frontiers Science Center for Nano-optoelectronics, Peking University, Beijing 100871, China

**Keywords:** silicon photonics, optical switch, reconfigurable optical interconnection, optical pipeline parallelism (OPP)

## Abstract

As artificial intelligence models continue to expand rapidly and evolve at an accelerating pace, the sustainability of scaling law faces challenges as computational resources cannot expand indefinitely. A more promising strategy is to use high-performance interconnections to compose multiple modest-capacity computing chips into a high-efficiency system, enabling comparable capability to that of much larger resource-intensive systems. However, an effective on-chip communication and network that provides high bandwidth, low latency, and large-scale parallelism to accelerate distributed computation is still lacking. Here, we address this challenge by proposing an on-chip, all-optical-interconnect-based hybrid optoelectronic distributed computing system, achieving two orders of magnitude lower inference latency than a single graphics processing unit while using only one-ninth of its computational resources. The 400-gigabits per second silicon photonic transceiver chips can provide high-speed and error-free optical input/output for processing chips, and an ultra-low-loss (*≤*5 dB at 1300 nm), non-blocking 16 *×* 16 optical switch chip can further scale out the all-optical network for massive and flexible interconnection. As a validation, we configure the system to realize optical pipeline parallelism and execute a five-layer convolutional neural network denoising model, which processes a total of 1000 images of 32 768 bits each in only 105.16 μs. These results convincingly illustrate a transformative pathway toward future high-efficiency computing architectures.

## Teaser

Harnessing OCS for scale-up AI networks enables ultra-low-latency, high-bandwidth distributed inference across chip-to-chip networks.

## INTRODUCTION

Fueled by the rapid development of artificial intelligence (AI) technologies, including large language models, diffusion models, and a growing variety of neural network architectures, the demand for computational resources has reached an unprecedented level in modern information society [1–4]. To meet this demand, a common approach is to follow the scaling law by improving the performance of processing chips such as graphics processing units (GPUs), while simultaneously scaling out the size of the computing cluster [5–8]. However, the recent studies on Deepseek-R1 model and Google Gemini3 model have achieved comparable performance through highly optimized algorithmic [9] and more efficient hardware architectures [10], indicating that specific objectives can be realized under limited computational resources when algorithms, processor micro‑architectures and chip‑level interconnections are co‑designed [11–14]. This fabric can also alleviate unsustainable energy usage in data centers and optimize latency or consumption in edge-computing scenarios [15–17].

In fact, AI workloads impose diverse demands [18,19]. Specifically, during the inference of deep neural networks (DNNs), numerous feature maps are generated between layers, posing challenges for temporary storage and management [20]. Despite parallel processors such as GPUs can prominently accelerate computation, the compute–memory separated Von Neumann architecture still leads to considerable latency due to the limited throughput between the processor and memory, known as the memory wall [21]. This issue becomes more severe in deep networks, where each layer must wait for the prior layer to complete and access data from memory, leaving the remaining layers idle and reducing computational efficiency. Therefore, in latency-sensitive edge computing scenarios, a more practical approach is to partition neural network layers across multiple processing chips, enabling pipeline parallelism [22]. The intermediate feature maps are directly transferred to the next chips for further processing, and all layers operate concurrently on continuous input streams, thereby reducing frequent memory access and fully utilizing computational resources. However, this architecture demands chip-level interconnection throughput to keep a stable, high-bandwidth data flow while eliminating processor stalls or pipeline bubbles [23].

Current electronic interconnect technologies face challenges in scaling up bandwidth and energy efficiency to support higher-throughput data flow, which has, in turn, become the primary catalyst for adopting optical interconnection [24–28]. Silicon photonics (SiPh) is widely acknowledged as a leading platform for optical transceivers in high-speed communication [29–33]. By further incorporating optical switch chips, optical data flows can be routed directly, avoiding additional electrical-to-optical (E-O) and optical-to-electrical (O-E) conversions, while the bufferless circuit-switched architecture enables low latency and high-throughput parallelism [34–37]. Nevertheless, realizing all-optical interconnection for DNNs inference acceleration, given the limited performance, remains far from practical. First, the optical switch chip should achieve both ultra-low insertion loss and an ultra-broad spectral response, which are essential to support wavelength-division multiplexing (WDM) to scale the aggregate data rate and the interconnect size. However, most prior works optimize insertion loss exclusively at a single wavelength, and only a few account for facet-coupling losses to ensure low fiber-to-fiber loss for practical applications. Second, introducing such an optical switch chip further imposes stringent requirements on the SiPh transceivers, because the additional insertion loss cannot be negligible compared with conventional low-loss fiber links [38,39]. Therefore, sustaining high-throughput interconnection under the low-SNR constraints of chip-level deployment requires further improvements in high-speed signal integrity. Finally, there is currently no demonstration of deploying practical AI workloads into such integrated all-optical scale-up networks with absolute latency advantages. This fundamentally requires dedicated algorithmic-hardware mapping, including adaptation to the physical-layer characteristics of all-optical links and efficient internal data scheduling on the computing chips.

In this work, we bridge these gaps by presenting an integrated hybrid optoelectronic distributed computing system, as illustrated in Fig. 1, which realizes the entire stack, from the physical-layer design of the underlying photonic chips to high-level inference algorithms. Each computing chip (implemented here using the field-programmable gate array, FPGA) is equipped with a SiPh transceiver, providing a high-speed optical input/output (I/O) interface of 400 gigabits per second (Gbps). A 16 *×* 16 non-blocking optical switch chip is implemented on a silica-on-silicon planar lightwave circuit (PLC) platform to scale up the interconnect network, featuring a record-low total loss of *≤*5 dB at 1300 nm over a nearly 100 nm spectral range. Experimental results confirm error-free 400 Gbps data transmission over the all-optical network without optical amplification. Based on this, we design a five-layer convolutional neural network (CNN) denoising model, deploy each layer onto a separate FPGA chip, and reconfigure the optical switch chip to establish the optical pipeline parallelism (OPP) among the five FPGA chips. Processing 1000 images of size 32×32×32 bits requires only 105.16 μs, with the deployed digital signal processor (DSP) resources delivering a total computing rate of about 1.969 TFLOPS. For comparison, the proposed system is nearly 149× faster than a single GPU while using only 0.116× of its total computational capacity. These results indicate that the high-throughput optical interconnect can fully release the available computational efficiency, and thereby pave the way for future ultra-low-latency edge-intelligent deployments.

**Figure 1.**
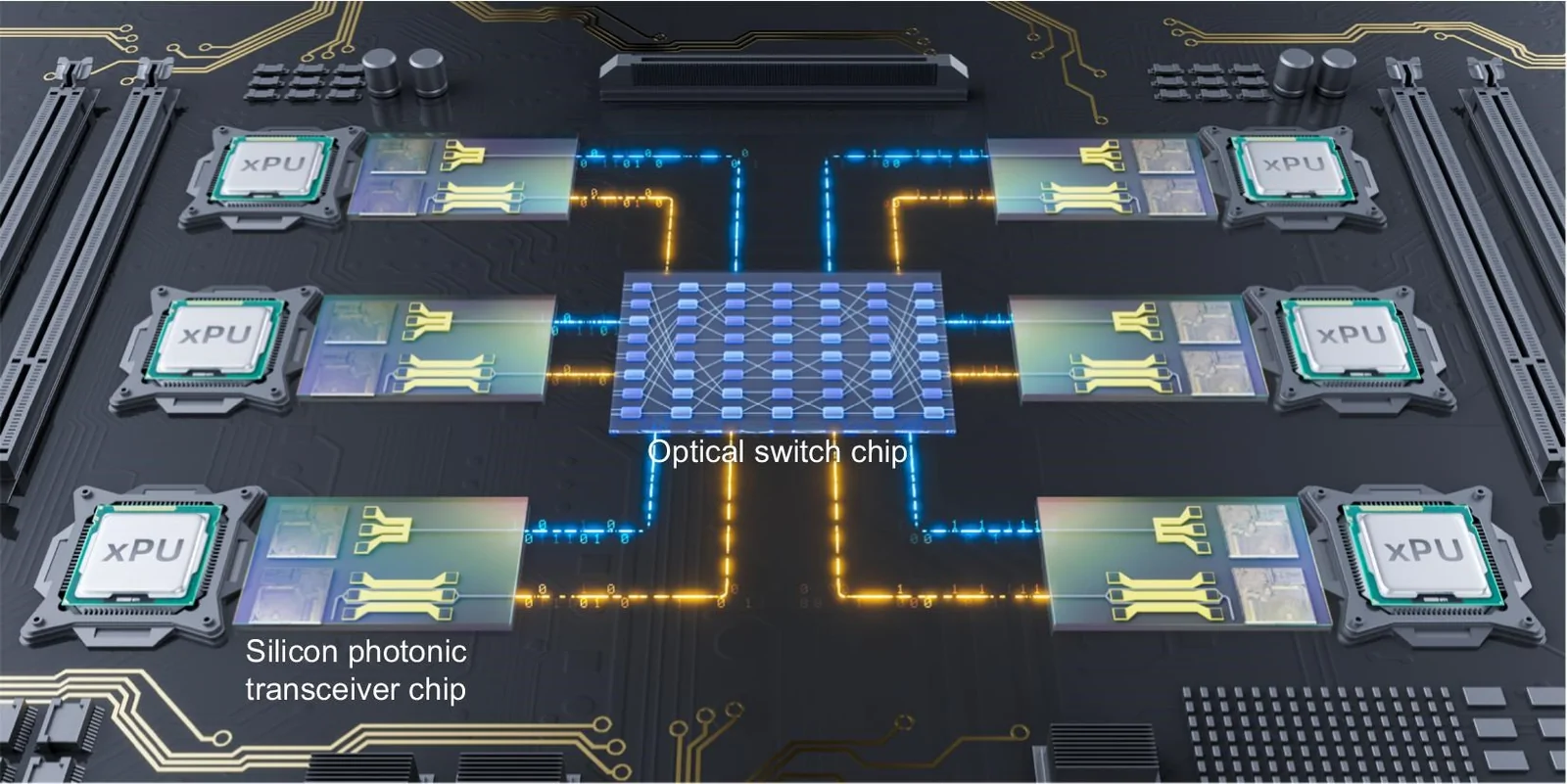
Conceptual illustration of the proposed integrated hybrid optoelectronic distributed computing system. The system can scale out to support up to 16 interconnected processing chips; for clarity, only a subset of the interconnect is shown.

## RESULTS

### Hybrid optoelectronic computing architecture

Figure 1 illustrates the proposed integrated hybrid optoelectronic distributed computing system, which consists of three key components: FPGA chips, SiPh transceiver chips, and a 16×16 optical switch chip. High-speed data flows are generated by an FPGA, transmitted through the silicon photonic I/O interfaces, routed by the optical switch chip, and ultimately received error-free by another FPGA. Together, these components form a reconfigurable, high-throughput computing fabric that interconnects up to 16 processor chips via the transceivers and the optical switch. This architecture implies that the optical switch chip can theoretically support up to 6.4 terabits per second (Tbps) of aggregate switching bandwidth, with the upper bound primarily limited by the output data rate of the FPGA.

The processors used in our demonstration are the commercially available FPGA chips (highlighted by the blue box in Fig. 2a). In addition to the basic logic elements and DSP units, it contains on-chip random-access memory (RAM) blocks, which are used here to buffer the I/O feature maps and store the kernel weights for computation. In addition, the FPGA provides 8 lanes of 50-Gbps high-speed serial I/O, with the physical media attachment (PMA) layer integrating essential signal-conditioning functions, including serial-in/parallel-out (SIPO), clock-data recovery (CDR), continuous-time linear equalization (CTLE), feed-forward equalization (FFE), and related circuitry. Furthermore, we designed customized development boards that host the FPGA. Each board integrates 16 GB dynamic RAM, thermal-management components, a peripheral component interconnect express (PCIe) interface for communicating with the server and initiating inference tasks, and a joint test action group interface for programming and debugging. It also features a high-speed electrical socket that ensures high-speed signal integrity for connection to the silicon photonic transceiver chip (highlighted by the red box).

**Figure 2.**
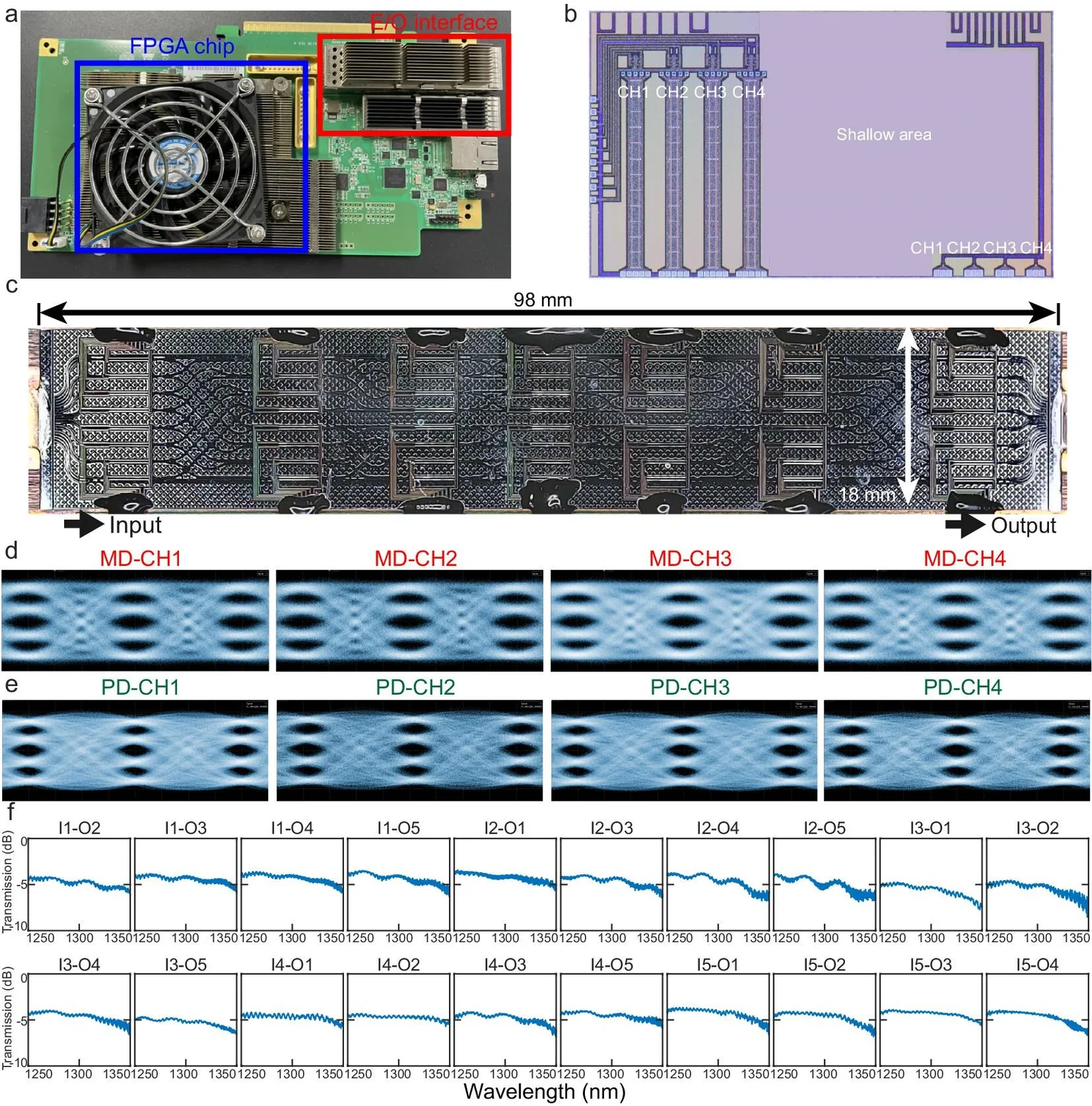
Optical images and performance characterization of the system components. (a–c) Optical images of the FPGA board (a), the silicon photonic transceiver chip (b), and the optical switch chip (c). The FPGA chip and the interface region where the photonic transceiver is connected with the FPGA's I/O are highlighted by rectangular outlines. (d, e) Measured 100-Gbps PAM4 eye diagrams from the four-channel modulators and photodetectors. (f) Representative fiber-to-fiber transmission spectra of the selected switching paths in the optical switch chip, shown after normalization to exclude losses from the external measurement setup. MD, modulator; PD, photodetector.

The SiPh transceiver is realized based on a four-channel traveling-wave dual-driver Mach–Zehnder modulator (MZM), and four-channel photodetectors (PDs), as illustrated in Fig. 2b. It was fabricated on a high-resistivity silicon-on-insulator (SOI) wafer with a 220-nm silicon device layer and a 2-μm buried oxide (BOX), using the complementary metal–oxide–semiconductor (CMOS) compatible process. Each MZM incorporates a PN junction whose p-type region is doped at $5\times 10^{17}$, the heavily doped p$^{+}$ region at $3\times 10^{18}$, and the ultra‑heavily doped P$^{++}$ region at $3\times 10^{20}\ {\rm cm}^{-3}$; the same doping profile is applied to the n-type region. A lateral P-i-N junction is implemented in each arm, positioned with a 35-nm horizontal offset from the waveguide center toward the n-type region, with an intrinsic region width of 30 nm. Intermediate P$^{+}$ and N$^{+}$ doping regions are positioned 300 nm away from the waveguide core edge to further decrease the series resistance of the PN junction while maintaining low optical loss. The P$^{++}$ and N$^{++}$ regions are placed approximately 605 nm away from the waveguide center to provide low-resistance ohmic contacts. The carrier-depletion MZM comprises two 3-mm active waveguides with 410-nm rib width and 70-nm slab thickness, delivering a 35 GHz E-O bandwidth and a half-wave voltage of 6 V. The PD used the vertical p-doped–intrinsic–n-doped (PIN) structure, implemented using germanium (Ge) epitaxial growth. The O-E bandwidth is up to 40 GHz with the responsivity of 0.8 A/W. Other details can be found in Note SI. Figure 2d shows the measured pulse-amplitude modulation with four levels (PAM4) eye diagrams for all MZM channels. All channels achieve 100-Gbps operation with the average transmitter and dispersion eye closure quaternary (TDECQ, which quantifies the optical power penalty caused by transmitter impairments compared to an ideal signal) of 2.05 dB and the average extinction ratio of 5.4953 dB. Corresponding PD eye diagrams (Fig. 2e) show clean openings at 100 Gbps with an average receiver TDECQ of 5.92 dB. During the execution of the computational tasks, the four-channel modulators and PDs operate at four distinct wavelengths: 1270, 1290, 1310, and 1330 nm. These four optical lanes are subsequently multiplexed into a single fiber using WDM technology and connected to the optical switch chip, as elaborated below.

**Figure 3.**
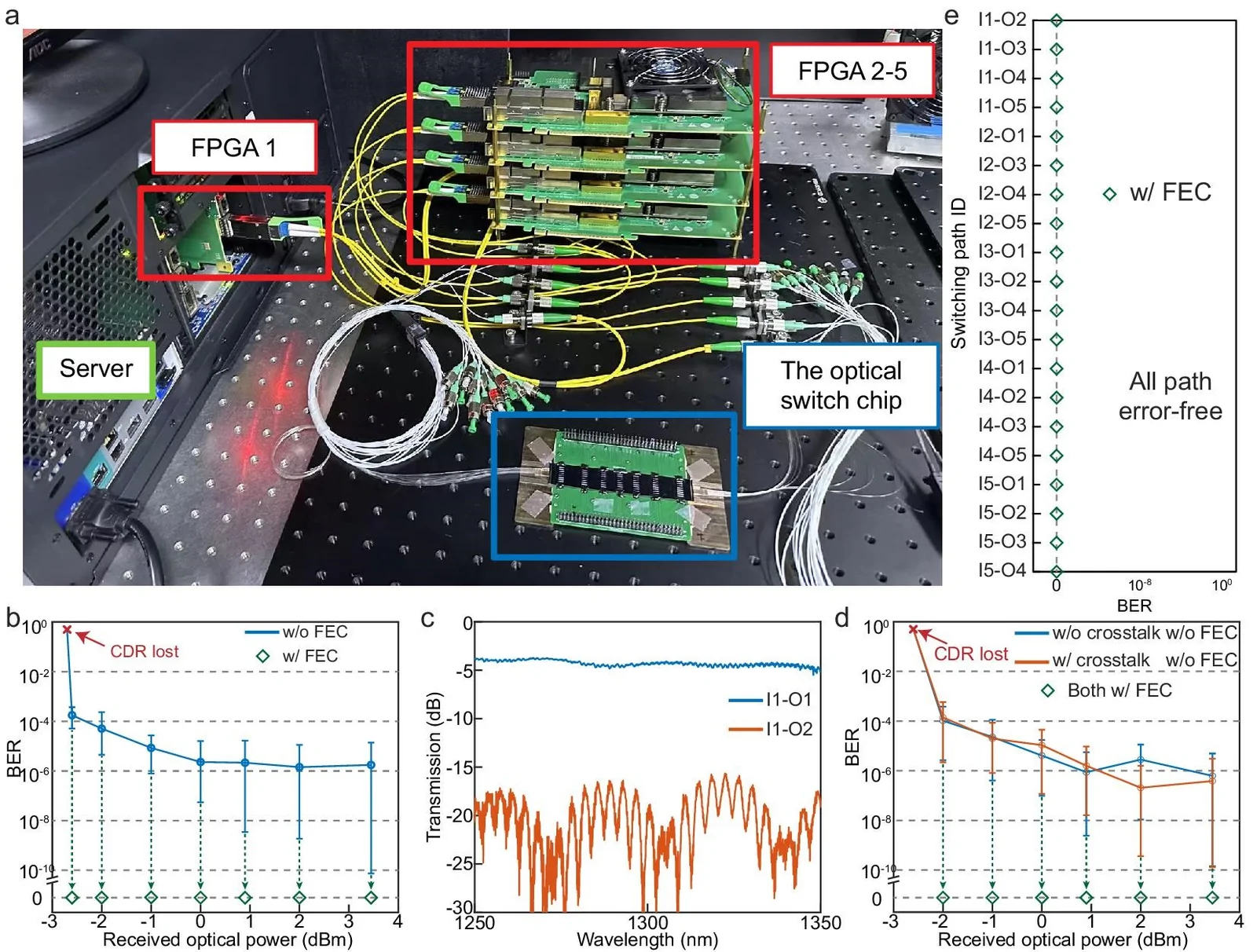
Quantitative characterization of the full-link communication performance. (a) Photograph of the full-link test setup, including a server, 5 FPGA boards with SiPh transceiver chips, and the optical switch chip. (b) Average bit error ratio (BER) measurements vs. received optical power for eight channels at the total rate of 400 Gbps. Error bars represent the observed minimum and maximum BERs of eight channels. (c) Transmission spectra of the selected switching path when the voltage bias corresponding to the I1–O1 path is applied. (d) Average BER measurements with and without crosstalk for eight channels at the total rate of 400 Gbps. Error bars represent the observed minimum and maximum BERs of eight channels. (e) BER measurement across 20 switching paths at 400 Gbps rate. FEC, forward error correction.

The 16$\, {\times }\, 16$ non-blocking optical switch chip is implemented using a Benes architecture [40], as illustrated in Fig. 1. The fabrication and other details can be found in Note SII. Figure 2c presents the optical microscope image of the fabricated switch chip, which has a footprint of 98 mm$\, \times \,$18 mm. Since a high-radix architecture requires multiple stages involving a large number of unit devices such as MZIs and waveguide crossings, it demands ultralow-loss characteristics to support such large-scale integration. The PLC platform effectively addresses this requirement due to its low index contrast. The edge couplers exhibit excellent mode matching with standard single-mode fibers, and the intrinsically low-loss silica waveguides further minimize on-chip insertion loss. Moreover, this low-loss performance can be maintained over a broad spectral range. As shown in Fig. 2f, the transmission spectra of the 16 *×* 16 optical switch chip were measured with both the input and output fiber arrays optically packaged to the chip. In the experiment, we selected 20 channels (from I1–O2 to I5–O4, which are used in the subsequent demonstrations) for wavelength-swept measurements from 1250 to 1350 nm. For almost all these channels, the total loss, including coupling loss and on-chip loss, is < 5 dB at 1300 nm, respectively. Moreover, the spectra exhibit excellent flatness across the entire 100 nm bandwidth, with the minimum peak-to-valley variation of 1.39 dB. This flat spectral response is critical for enabling WDM in the proposed system. Wavelength-dependent loss is difficult to equalize with conventional optical amplifiers. If low insertion loss cannot be maintained over a sufficiently broad wavelength range, certain channels will experience excessive attenuation, leading to increased bit errors or even loss of clock-and-data-recovery (CDR) lock.

This directly affects the port utilization efficiency of the switch. Using our four-channel SiPh transceiver as an example, if the switch bandwidth were too narrow to support WDM, a single FPGA would have to occupy four physical switch ports, one per wavelength channel. Under this constraint, a 16 *×* 16 switch could interconnect at most 4 FPGA chips, limiting the total aggregate switching bandwidth to 1.6 Tbps. In contrast, the flat, broadband response of our optical switch allows the four wavelength channels to be multiplexed onto a single fiber/waveguide, so each FPGA occupies only one switch port. This increases the supported number of interconnected computing chips to 16 and raises the maximum aggregate switching bandwidth to 6.4 Tbps.

### Error-free reconfigurable all-optical interconnection

We further package the SiPh transceiver chip into the FPGA-board socket with a serdes chip for signal de-/serialization, and interconnect five FPGA chips via the optical switch chip to form a distributed computing system, as illustrated in Fig. 3a. Further details of the package can be found in Note SI. The exceptionally broadband spectral response of the optical switch chip makes it an ideal match for WDM technology. Consequently, optical data flows from multiple channels can be multiplexed onto a single fiber, allowing each FPGA chip to occupy only one port with a communication rate of 400 Gbps. Additional wavelengths can further increase the capacity, up to the limit imposed by the effective spectral bandwidth of the optical switch chip.

To evaluate the fundamental chip-to-chip link performance, we interconnected two FPGA boards in a bidirectional configuration through the optical switch. One FPGA generated a 400-Gbps pseudo-random binary sequence of order 31 (PRBS31), which was transmitted through the all-optical network and then received and decoded by the other FPGA. An optical attenuator was inserted in the link to assess the optical power budget of the experimental setup. As shown in Fig. 3b, the minimum BER without additional attenuation is $4.34 \times 10^{-10}$. We then increased the attenuation to measure the BER as a function of the received optical power at 400 Gbps. When the additional attenuation reached 6.15 dB, the CDR integrated in the receiving FPGA could no longer acquire lock to recover valid signals. Moreover, as indicated by the green diamond markers and green dashed line in Fig. 3b, once the FPGA’s built-in FEC is enabled, the received signal can remain error-free until the CDR loses lock. In conclusion, only about 11–12 dB of the overall link power budget can be allocated to the optical switch chip. To the best of our knowledge, this represents the first quantitative characterization of link-budget requirements in the chip-to-chip communication system.

We further investigated the impact of crosstalk between adjacent channels in the optical switch chip. As illustrated in Fig. 3c, when the switching path I1–O1 was activated by applying a voltage bias, we measured the transmission spectra of both I1–O1 (blue curve) and its adjacent path I1–O2 (red curve), obtaining an extinction ratio of 16.45 dB at 1300 nm. Then, to evaluate the impact on communication performance, we first measured the BER with a single path activated (blue curve), and subsequently activated two paths simultaneously to measure the BER (orange–red curve), as shown in Fig. 3d. The measured BER curves show no obvious difference between the cases with adjacent channels activated or not, confirming that the additional crosstalk introduced by the optical switch chip remains in an acceptable range for high-speed communication.

Finally, we demonstrated the reconfigurability of the hybrid optoelectronic distributed system. By tuning the voltage bias on the TiN heater to reconfigure the internal routing of the optical switch chip, arbitrary point-to-point routes could be reconfigured in the optical switch chip, establishing any desired connection among the five FPGAs. As shown in Fig. 3e, we tested the switching path IDs from I1–O2 to I5–O4 (excluding self-loopback such as I1–O1) and measured the BER under FEC-enabled conditions. All 20 tested routes exhibited error-free performance, enabling the use of more efficient data scheduling algorithms without the need for retransmission mechanisms that would otherwise introduce additional latency overhead.

**Figure 4.**
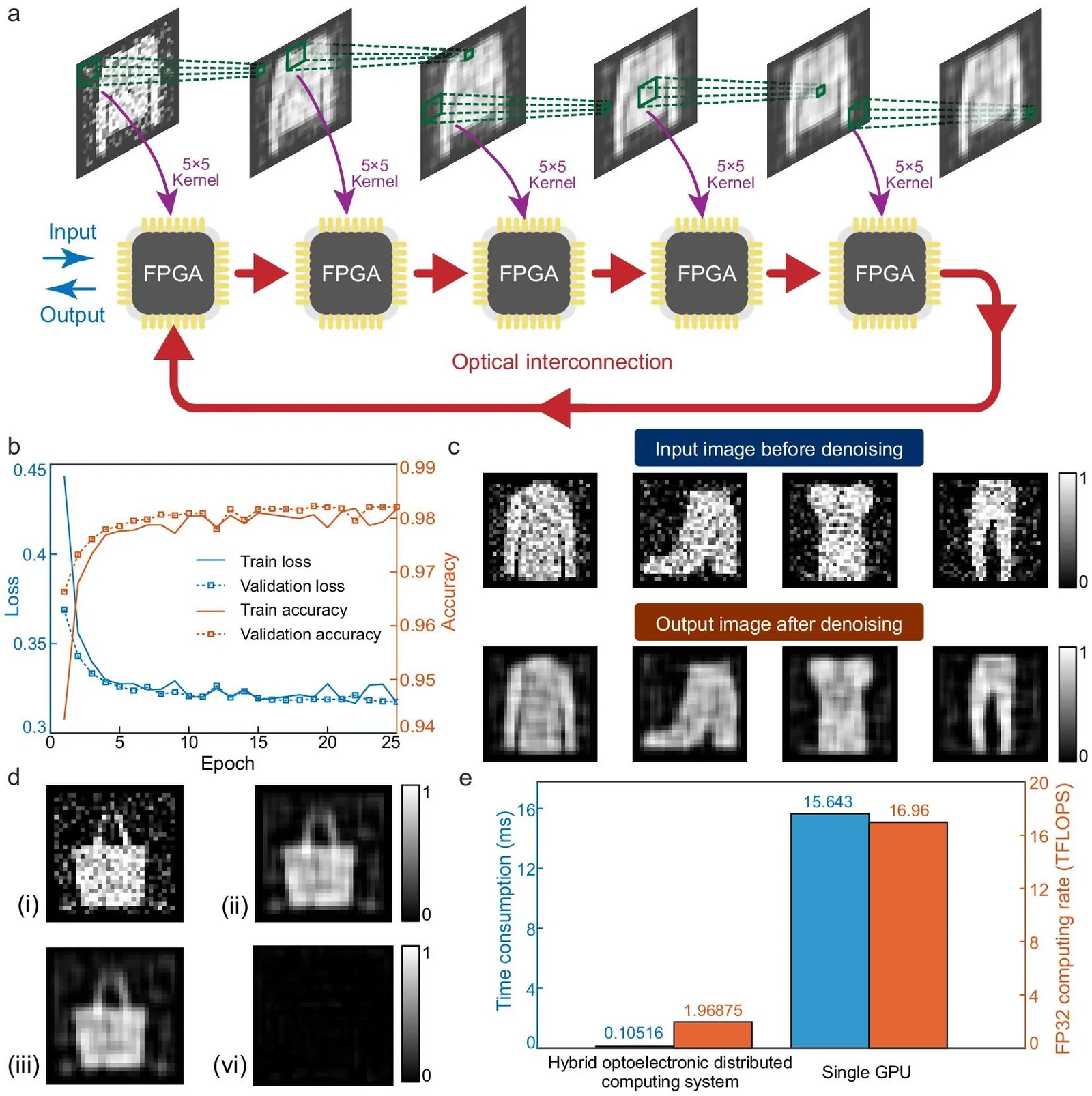
CNN-based image denoising task. (a) Schematic of the proposed system executing the 5-layer CNN image denoising task. The optical switch chip is reconfigured to form an OPP to link the five FPGA chips. (b) Training and validation loss and accuracy curves. (c) The image before (up panel) and after (bottom panel) denoising. (d) Representative results showing: (i) the input image before denoising; (ii) the image denoised by the proposed system; (iii) the image denoised by a single GPU; and (iv) the pixel-wise difference between (ii) and (iii). (e) Comparative analysis of inference latency and computational resources between the proposed system and a single GPU.

### High-throughput, OPP accelerating distributed inference computation

After validating that the all-optical interconnection via the SiPh transceiver chips and the optical switch chip provides high-speed and stable error-free chip-to-chip communication, we then performed experiments to demonstrate the capability of accelerating distributed inference computation. We set the internal routing to realize an OPP among the five FPGAs through the switching paths I1–O2, I2–O3, I3–O4, I4–O5, and I5–O1. The link topology and computational principle are illustrated in Fig. 4a. We constructed a five-layer fully CNN for the image denoising task on the Fashion-MNIST dataset. Each input image has a resolution of 32×32 pixels, with every pixel represented in 32-bit floating-point format, corresponding to 32 768 bits per image. Each layer consists of a 5×5 convolutional kernel followed by a rectified linear unit (ReLU) activation, forming a strictly symmetric topology. The resulting 28×28 feature map is then zero-padded back to 32×32 pixels. We then partitioned the five-layer neural network (convolutional encoder 1, convolutional encoder 2, convolutional decoder 1, convolutional decoder 2, and convolutional decoder 3) onto the five FPGAs in a layer-by-layer manner. During computation, each FPGA chip allocated an on-chip RAM buffer to store the incoming intermediate feature maps, which were subsequently sent to the DSP arrays for convolution with the kernel weights. The output of each stage was directly transferred to the next FPGA through the all-optical interconnection, effectively avoiding the memory-wall limitation. More details can be seen in Note SIII. To enable convenient measurement of the inference latency during the experiment, the setup was designed as follows. The first FPGA communicated with the server via a PCIe interface, and was responsible for initializing the inference task, loading the dataset, and reporting the final results. The final results from the fifth FPGA were returned to the first along the I5–O1 path. The total inference latency was measured by an internal timer on the first FPGA, which stopped only after all results from the fifth FPGA had been fully received. Consequently, the reported runtime is slightly longer than the pure inference time, because in a real-world deployment, the task would be considered complete as soon as the fifth FPGA finishes its computation.

Figure 4b shows the accuracy and loss curves of the model, which was pre-trained offline outside the proposed system and exhibits good convergence. Figure 4c shows the result of the image denoising from the Fashion-MNIST dataset. Compared with the initial image dataset, the reconstructed image exhibits visibly reduced noise while preserving the key structural details. Fine textures and edges are effectively restored, demonstrating the effectiveness of the distributed computing results. To further verify that the high-throughput optical interconnection does not introduce any unintended artifacts into the computation, we executed the same task on a commercially available GPU for comparison. As shown in Fig. 4d, Panels (ii) and (iii) display the inference results from the distributed FPGA system and the GPU, based on the same input image obtained, respectively. Panel (iv) presents the pixel-wise difference between the two outputs. The difference map is identically near-zero across all pixels, confirming that the transmission through the optical interconnection preserves numerical integrity and introduces no errors.

Finally, we executed 1000 images denoising task with the size of 32×32 pixels using this integrated hybrid optoelectronic distributed computing system, and compared the latency with that of a single GPU, as shown in Fig. 4e. Both systems utilize 32-bit floating-point (FP32) precision for all computations. More details of GPU baseline configuration and benchmarking methodology can be found in Note SIV. For the hybrid optoelectronic distributed computing system, the total latency was measured as 105.16 μs. During computation, the images were grouped in batches of 10, streamed into the FPGA’s DSPs for convolution, and then forwarded to the next FPGA chip in the pipeline. Given that the proposed CNN model utilizes a total of 5040 DSPs operating at a 390.625 MHz clock frequency, the corresponding peak 32-bit floating point (FP32) computing rate is estimated as $5040 \times 390.625\ {\rm MHz} = 1.96875$ tera floating-point operations per second (TFLOPS). For an input dataset of 1000 images, the total FP32 operations (N) is calculated to be 196 mega floating-point operations (MFLOPs), as given by the equation below:


(1) \begin{eqnarray*}
\mathrm{N} = H \times W \times K_h \times K_w \times 2 \times C_l \times 1000,
\end{eqnarray*}

where *H* and *W* denote the spatial resolution of the feature maps (both equal to 28), $K_h$ and $K_w$ represent the kernel dimensions (5×5), and $C_l$ indicates the total number of layers/FPGA chips, which is 5. Based on the above formulation, the theoretical processing latency can be estimated under an ideal full-utilization assumption, where the DSP resources on the FPGA execute the FP32 operations continuously without idle cycles. Under this assumption, the theoretical latency is 99.6 μs. Compared with this bound, the measured latency corresponds to a compute-utilization efficiency of 94.7%, indicating that the system operates close to the theoretical optimum due to the high efficiency OPP. We further evaluated a commercially available GPU as a baseline for comparison. The GPU integrates 3072 CUDA cores capable of executing fused multiply–add operations. When processing this same computation workload, it operated at a clock frequency of 2.76 GHz, resulting in a theoretical peak FP32 computing rate of approximately 16.96 TFLOPS. However, the measured execution time on the GPU is 15.643 ms. Such performance degradation can be primarily attributed to the memory wall. After completing each layer, the GPU must temporarily store the intermediate feature maps in external memory. It then reconfigures the next layer’s weights/kernels before fetching the stored feature maps back as inputs for the subsequent computation. This procedure implies that, at any given moment, the GPU can only process one layer, significantly limiting parallelism compared with the proposed system.

## DISCUSSION AND CONCLUSION

The current demonstration employs only 5 *×* 5 convolution kernels, which represents a relatively small workload compared by modern AI standards. The primary limitation arises from timing constraints of the programming workflow inside the FPGA, which could be alleviated through further optimization. Even so, at the current system scale, compute-unit utilization remains below 97%, indicating the presence of residual idle cycles in the DSPs. This suggests that the utilization can be further improved by scaling up the interconnection throughput to fully drive the available computational capacity. Notably, this bottleneck originates from the limited I/O bandwidth of the FPGA chips rather than the silicon photonic transceivers, which already support per-wavelength data rates of 200 Gbps and aggregated capacities of up to 1.6 Tbps [41,42]. These results underscore the need for future processing chips to scale both I/O channel count and bandwidth to meet the demands of large-scale AI workloads.

In this experiment, data communication among the FPGA chips uses a standard Ethernet protocol, which is generally regarded as higher-latency than specialized chip-to-chip protocols such as PCIe or InfiniBand [43]. However, in our proposed system, the latency overheads associated with full Ethernet protocol stacks are removed. Correspondingly, the functions of switching and re-routing are handled by an optical switch chip, which is a passive waveguide fabric with no packet scheduling, queuing, or buffering. As a result, the photonic transceivers and the optical switch together provide the interconnection function that operates at the physical layer for extremely low latency. Only the essential data-encoding schemes, such as 64b/66b encoding and FEC, are retained. Moreover, adopting the Ethernet protocol can also eliminate the need to translate out-of-band control signals into the optical domain, such as the PCIe protocol, preserving a concise data-link pathway for chip-to-chip communication.

Meanwhile, the effective net throughput of the optical interconnection measured in the experiment is slightly below 400 Gbps, due to the remaining protocol-encoding overhead and the use of FEC to compensate for signal impairment. Therefore, more advanced packaging approaches such as co-packaged optics are highly desirable as the electrical path between the processing chip and the photonic transceiver can be significantly shortened to improve the signal quality. In this way, the system could operate with reduced overhead, thereby improving the effective net throughput and enabling higher chip-to-chip communication performance for large-scale distributed computing.

## Supplementary Material

nwag282_Supplemental_File
