# Supplementary material for: On-chip large-scale all-optical interconnect for ultra-low-latency deep neural network inference
Source: Natl Sci Rev. 2026 May 19;13(19):nwag282. doi: 10.1093/nsr/nwag282 (PMC13637597; doi:10.1093/nsr/nwag282)
Supplement: Supplementary file 1 [file nwag282_supplemental_file.pdf]

Supplementary Information for

On-chip large-scale all-optical interconnect for ultra-low-latency deep neural network inference

Supplementary Note I: High-speed interconnect optical and electrical link

The light source within the transceiver is a 4-channel distributed feedback (DFB) laser array, operating at wavelengths of 1270nm, 1290nm, 1310nm, and 1330nm, respectively. These four wavelengths are assigned to the four parallel traveling-wave Mach-Zehnder modulators (MZMs) on our chip. The laser power of each channel is 100 mW. The silicon photonic (SiPh) chip includes four Mach–Zehnder modulators (MZMs), each coupled to one channel of the DFB laser array. The coupling loss of each channel is about 2 dB. The FPGA chip provides eight lanes of high-speed serial I/O, each operating at a maximum data rate of 50 Gbps. Physically, a single lane corresponds to a differential pin pair (transmitting positive and negative high-speed signals) that routes the electrical data off-chip. From the FPGA differential pins, the eight 50-Gbps signals propagate along a short stretch of printed circuit board (PCB) traces to the high-speed socket for E-O/O-E conversion. Here, we strictly limit the routing length between the FPGA and the high-speed connector to maximize signal integrity. This approach directly aligned with the motivation of Co-Packaged Optics (CPO) and Near-Packaged Optics (NPO), which is closely integrated with the electrical chip, thereby shortening high-speed electrical links and reducing loss and distortion.

In the socket assembly, the signals first pass through a DSP chip equipped with serializer/de-serializer (SerDes) functionality (also referred to as a gearbox in some situations), which multiplexes pairs of the 50-Gbps electrical lanes into four 100-Gbps data streams. These four pairs of 100-Gbps differential electrical signals are then amplified to approximately 2.75 Vpp by the DSP's internal driver and routed to the electrical pads of the four MZMs. The output modulated optical signals of the MZM are coupled out through the edge coupler. An off-chip wavelength division multiplexing device will be used to combine the 4-channel optical signals (output power of each channel is approximately 2 mW). The optical signals are routed to the receiver through an optical switch chip exhibiting an approximate insertion loss of 5-6 dB, depending on the specific channel and wavelength.

At the receiving part of each transceiver, there is a demultiplexing device used to separate the optical signals of four wavelengths. The four-channel GeSi detector array on the SiPh chip converts optical signals into current signals, which are then converted into voltage signals by a TIA and transmitted to the DSP for further processing. The optical power received by each GeSi detector is approximately 300 μW, the converted current signal is approximately 200 μA, and the voltage converted by TIA is approximately 500 mVpp. After the electrical signals are re-timed and equalized by

DSP, the signal will be transmitted to the FPGA. The consumption of the SiPh transceiver is around 10 W.

Fig.S1b illustrates the diagram of the data link within FPGA chips from the logic elements to the high-speed I/O pins. In the transmit direction, data generated in the programmable logic elements of the FPGA core passes through the internal routing to the transceiver die. The data then enters the Ethernet Hard IP block, which includes the media access control (MAC) and physical coding sublayer (PCS). The MAC layer handles data framing and addressing, and the PCS performs data scrambling and block alignment. Next, the data goes to the forward error correction (FEC) engine, which is a fixed hardware circuit. The FEC adds parity bits to detect and correct errors during transmission. With this FEC overhead included, our measured net data throughput rate is approximately 360 to 370 Gbps. After the FEC, the data moves to the physical medium attachment (PMA). The PMA serializes the parallel data and modulates it into 8 lanes of 50 Gbps PAM4 signals. These signals are then output through the TX pins to the optical transceiver. In the receive direction, the received 8 × 50 Gbps electrical signals enter the RX pins and go to the RX PMA for clock and data recovery (CDR) and deserialization. The data then flows through the hardware FEC block for error correction, passes through the PCS and MAC, and passes back to the FPGA core logic.

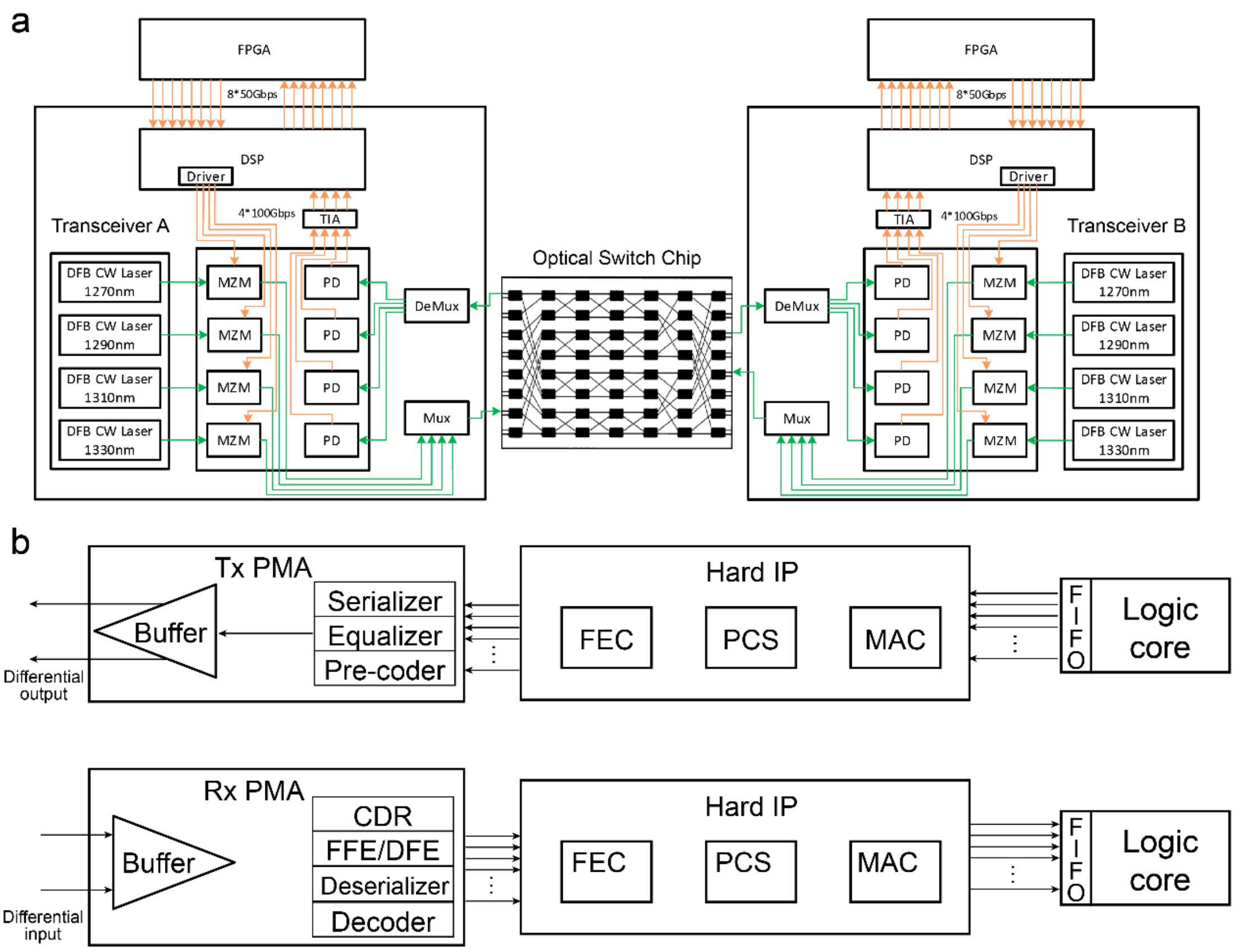

Fig.S1: a, Optical and electrical link diagram. b, diagram of the FPGA's data link from the logic elements to the high-speed I/O pins

Supplementary Note II: The fabrication details and performance analysis of the optical switch chip

A PLC-based 16×16 Mach–Zehnder interferometer (MZI) optical switch matrix for O-band operation (design wavelength 1295 nm; nominal index contrast 2%; 3×3μm waveguide cross-section) is fabricated on a silica-on-silicon stack using a fixed process flow. The process starts with the formation of a thick SiO_2 buffer layer on the silicon substrate by thermal oxidation. A Ge-doped silica core film is then deposited by plasma-enhanced chemical vapor deposition (PECVD) and annealed to stabilize the glass network and optical properties. Waveguide patterning is performed by standard photolithography, followed by dry etching to transfer the pattern into the deposited glass. Reactive ion etching (RIE) is used to obtain the anisotropic sidewall profiles required for waveguide definition in relatively thick silica layers. Before the core etch, a hard-mask layer is grown. The resist-defined pattern is transferred into a polycrystalline silicon (poly-Si) hard mask, and the resist is subsequently stripped. The Ge-doped core is then etched by RIE using the poly-Si mask, after which the poly-Si mask is removed. After the waveguides are defined, a boron- and phosphorus-doped silica upper cladding is deposited by PECVD. The cladding is densified through multiple anneal cycles to fully bury the waveguides and to improve step coverage. Dopant incorporation during PECVD provides refractive-index engineering: Ge increases the refractive index, whereas phosphorus (P) and boron (B) are used to tune the refractive index and reduce glass viscosity, which benefits gap filling and topography smoothing. Thermo-optic phase control is then implemented using lithography-defined lift-off metallization. The heater layer is formed by sputter deposition and lift-off, with a heater width of 21μm. Electrical routing and interconnect metallization are subsequently formed by a second lift-off process based on metal evaporation. Thermal management is completed by deep etching of thermal-isolation trenches. The trenches are defined with a 35 μm width and a 13 μm spacing from the waveguides to suppress lateral heat spreading and reduce thermal crosstalk in the dense MZI array. The required trench depth is obtained by dry etching through the silica stack and, where necessary, extending the etch into the silicon substrate under hard-mask–assisted RIE conditions to maintain depth and profile control. Through systematic optimization of film deposition and densification, pattern transfer, dry-etch profile control, and thermo-optic isolation, this process flow enables low-loss buried waveguides and stable thermal phase tuning, thereby supporting a broadband, low-insertion-loss optical switch matrix suitable for O-band operation.

As for the structure, the proposed optical switch chip comprises 56 cascaded 2×2 MZ

interferometers (MZIs). Each MZI is constructed with an input 3-dB directional coupler, two independent interferometric arms, and an output 3-dB directional coupler. A TiN heater is placed on top of each arm to tune the local temperature, thereby modifying the refractive index of the underlying waveguide material and enabling precise control of the differential phase shift between the two arms. The optical switch chip is fabricated on a silica-on-silicon PLC platform, where a high-index buried waveguide core is embedded within lower-index upper and lower cladding layers. By engineering the waveguide doping, the refractive-index contrast between the core and cladding is set to 2%.

To realize signal routing, the proposed optical switch chip relies on cascaded 2×2 MZ interferometers (MZIs). By applying a voltage to the waveguide micro-heaters, the thermo-optic effect induces a phase shift that alters the interference condition, thereby dynamically setting the output splitting ratio. In our experiment, the average power consumption required to establish a single optical routing path is approximately 1.789 W. Regarding the reconfiguration speed, the response time is primarily governed by the thermal dissipation rate of the silicon waveguide. The switching frequency of the thermo-optic effect typically falls in the tens of kilohertz range, corresponding to a reconfiguration time on the order of tens to hundreds of microseconds. In the future, to further optimize the optical switch, both power consumption and reconfiguration speed can be significantly improved with advanced device technology. Specifically, introducing thermal isolation structures, such as air trenches or suspended waveguides [3], can effectively minimize heat dissipation to the silicon substrate, thereby reducing the required thermal tuning power. Meanwhile, to achieve nanosecond-scale reconfiguration speeds, the new physics mechanism can be employed. For example, recent breakthroughs in parity-time (PT) symmetric optical switches have demonstrated ultrafast switching dynamics down to the 100 ps scale by dynamically tuning optical gain and loss [4].

Supplementary Note III: FPGA-Based OPP implementation and scalability analysis

To implement the image denoising task, we adopted a five-layer deep CNN, where each layer contains a single 5×5 convolution kernel. Following the architecture of the hybrid optoelectronic system, each convolutional layer was deployed onto a separate FPGA, forming a pipelined computing architecture. The five convolution kernels are listed as follows:

$$C_1 = \begin{bmatrix} 0.0168 & -0.0131 & 0.0492 & 0.0017 & -0.0289 \\ 0.0213 & -0.0020 & 0.1381 & 0.0089 & -0.0142 \\ 0.0500 & 0.1273 & 0.3660 & 0.0861 & 0.0052 \\ 0.0288 & 0.0133 & 0.1238 & 0.0076 & -0.0264 \\ 0.0113 & -0.0189 & 0.0464 & -0.0014 & -0.0444 \end{bmatrix} \quad (1)$$

$$C_2 = \begin{bmatrix} -0.1117 & 0.0358 & 0.0180 & -0.0900 & 0.0509 \\ -0.0327 & 0.0223 & 0.0751 & -0.0106 & 0.0207 \\ -0.0186 & 0.0733 & 0.5282 & 0.3170 & 0.0465 \\ 0.0022 & -0.1354 & 0.3270 & -0.1802 & 0.0988 \\ -0.1146 & 0.0840 & -0.0746 & 0.0077 & 0.0520 \end{bmatrix} \quad (2)$$

$$C_3 = \begin{bmatrix} 0.0674 & -0.0364 & 0.0976 & -0.0050 & -0.0915 \\ 0.0082 & -0.0691 & 0.1169 & -0.0051 & -0.0119 \\ 0.0477 & 0.1815 & 0.5781 & 0.1225 & -0.0323 \\ 0.0232 & -0.0649 & 0.2161 & -0.0936 & -0.0013 \\ -0.0133 & -0.0136 & 0.0973 & -0.0253 & -0.1127 \end{bmatrix} \quad (3)$$

$$C_4 = \begin{bmatrix} -0.0031 & -0.2422 & 0.2253 & -0.1211 & -0.0064 \\ -0.1035 & 0.0663 & 0.3570 & -0.1797 & 0.0670 \\ 0.0885 & 0.2247 & 0.2527 & 0.3799 & -0.0361 \\ -0.0449 & 0.1355 & 0.0247 & -0.1127 & 0.0491 \\ -0.0054 & -0.2102 & 0.2131 & -0.0024 & -0.0225 \end{bmatrix} \quad (4)$$

$$C_5 = \begin{bmatrix} 0.0601 & -0.0310 & 0.1017 & -0.0768 & 3.21 \times 10^{-4} \\ -0.2134 & 0.1217 & 0.0142 & -0.0863 & 0.0609 \\ 0.2661 & 0.0219 & 0.4508 & 0.3907 & -0.0943 \\ 0.2007 & -0.0964 & 0.3624 & -0.0033 & -0.0626 \\ 0.1101 & -0.0535 & 0.1034 & -0.2304 & 0.0873 \end{bmatrix} \quad (5)$$

Each FPGA executes one convolutional layer and constructs a high-efficiency processing structure that enables coordinated scheduling between data flow and computational resources. Specifically, six DSP blocks are grouped into one processing element (PE). Five DSPs operate in parallel to receive five input pixels and their corresponding convolution weights for multiplication, and the sixth DSP performs accumulation. In this way, the PE completes two vector multiply and accumulate operations on 1×5 vectors. An array of 28 by 6 PEs was constructed on each FPGA to support parallel convolution. After zero padding, each input image becomes a 32×32 pixel matrix, requiring 28×28 convolution operations. Since the PE array has 28

rows, an entire column of the image can be processed simultaneously. Within each row, the six PEs compute convolutions on columns 1 through 6 of the image at the same time. As a result, 28 by 6 convolution operations are completed in six clock cycles, with five cycles used for vector multiplication and one cycle for accumulation. A full convolution of one image is completed in 30 clock cycles. Through this layer-wise partitioning and the scheduling of data flow, the system achieves highly parallel computation and efficient use of available compute resources.

In the hardware implementation, the forward error correction (FEC) module within the FPGA transceiver is enabled to maintain error-free optical links. Therefore, the measured end-to-end latency of 105.16 μs already includes these overheads, such as serialization and deserialization (SerDes), electro-optic conversion, fiber propagation delay, and FEC encoding/decoding. The standalone latency of the FEC blocks is on the order of hundreds of nanoseconds. Because the system employs pipeline parallelism, this FEC delay only increases the initial pipeline fill time. It does not accumulate at each routing node. Once the pipeline stages are fully loaded, the optical transmission and FEC decoding of incoming data overlap entirely with the ongoing matrix computations. Under a continuous data stream, the distributed network effectively operates with a single communication hop. Therefore, the overall system latency is determined by the optical I/O bandwidth, making the specific FEC processing time negligible.

The current scale of the five-layer CNN with 5×5 convolution kernels presented in the main text is not constrained by the hardware limitations of the proposed hybrid optoelectronic distributed system. FPGA resource utilization reports indicate that the current implementation occupies approximately 12% of the available DSP blocks and 23% of the on-chip memory resources, leaving 88% of DSP resources and 77% of memory capacity unused. Therefore, the demonstrated network scale does not approach the hardware capacity limit of the platform. To deploy deeper neural networks, we can scale up the system by cascading more FPGA chips based on the proposed scalable optical interconnection architecture. Currently, the optical switch chip implemented in our system has 16 ports, supporting up to 16 interconnected computing chips. This scale can be further extended by adopting a larger-port-count switch based on the same design architecture. Scaling toward wider networks, such as those with increased channel counts or larger feature maps, can be realized by more fully utilizing the remaining DSP and memory resources within each FPGA. This requires more advanced FPGA design optimization, including refined parallelization strategies, routing management, and timing closure. The current level of resource utilization indicates substantial remaining capacity for the implementation of larger convolutional

layers and more computationally intensive operations.

In addition, more complex neural network architectures, including residual connections, skip connections, and attention mechanisms, can be supported through the flexible and reconfigurable inter-chip routing enabled by the optical switching fabric. The dynamic optical interconnection allows arbitrary communication patterns between distributed compute chips, enabling structured dataflows beyond strictly feedforward pipelines.

Furthermore, we also provide an additional discussion here on how interconnection bandwidth and memory bandwidth affect the overall performance of the proposed system. Further increasing the interconnection bandwidth can improve the system scale and overall performance. In the current architecture, doubling the batch size per chip from 10 to 20 images, or doubling the pixel count of each image, would proportionally double the required optical bandwidth to approximately 800 Gbps. At present, the optical interconnection operates at 400 Gbps, primarily limited by the electrical I/O bandwidth of the FPGA rather than by the optical switch or the SiPh transceiver itself. Therefore, future scaling of FPGA I/O bandwidth can directly improve the interconnection bandwidth. As for the impact of memory bandwidth, since the adopted pipeline parallelism minimizes repeated off-chip memory access, the time consumption of memory access will not become a limitation if the memory bandwidth can match the interconnect I/O bandwidth. This is quite easy to ensure a high bandwidth of memory, as the memory chips are usually placed very close to the computing chip, which reduces transmission loss and the parasitic effects for high-speed signals. In fact, such architecture like pipeline parallelism is a common strategy for latency-sensitive edge-computing scenarios to significantly reduce memory access overhead. To conclude, the five-layer CNN serves as a proof-of-concept workload to validate the end-to-end hybrid optoelectronic distributed architecture. The proposed system does not impose intrinsic limitations on network depth, width, or architectural complexity, and can be extended to larger-scale neural networks with appropriate FPGA design optimization and system-level scheduling.

As for power consumption, the silicon photonic transceiver consumes approximately 10 W, and the optical switch chip consumes 8.9 W to maintain five optical paths. In contrast, the GPU benchmark reports a power consumption of 55.34 W. A strictly fair comparison between these heterogeneous architectures is challenging: a GPU-based platform typically includes additional system power (e.g., motherboard and CPU), whereas the proposed hybrid optoelectronic prototype should also include the FPGA power. More importantly, the appropriate metric for comparison is the total energy required to complete the same task (in joules), which depends on both power and

execution time. Since our demonstration requires two orders of magnitude less execution time than the GPU-based system, the corresponding energy advantage is expected to be even more pronounced. In the future, the adoption of CPO/NPO architectures can further reduce power consumption because shortening the distance between the silicon photonic transceiver and the FPGA improves high-speed signal integrity and reduces electrical loss, thereby lowering the power required by the associated high-speed I/O blocks. We expect this to be a clear technology direction and to further strengthen the advantages of optical interconnects in chip-level deployments.

Supplementary Note IV: GPU baseline configuration and performance characterization

The five-layer CNN used in this work was trained offline on the Fashion-MNIST dataset using TensorFlow 2.10.0 (Python 3.8.20) with GPU acceleration enabled on an NVIDIA GeForce RTX 4060. Following standard supervised denoising training, Gaussian noise was injected into the normalized input images, and the network was optimized to reconstruct the corresponding clean targets via backpropagation. Model parameters were updated using the Adam optimizer with the binary cross-entropy objective, and training progress was monitored over 25 epochs using both the training loss and validation loss, together with a denoising accuracy metric defined as 1−MSE.

For the GPU baseline comparison, inference experiments were performed on an NVIDIA GeForce RTX 4060 GPU with a theoretical peak FP32 performance of 16.96 TFLOPS. The experimental environment was configured with Python 3.8.20 and TensorFlow 2.10.0, accelerated by CUDA Toolkit 11.8 and cuDNN 8.7.0. The identical five-layer CNN architecture was implemented on the GPU in FP32 precision. A total of 1,000 input images (32×32 pixels, 32-bit floating-point format) were used as the inference workload.

To determine an appropriate batch configuration, we evaluated different batch sizes while keeping the total number of input images fixed at 1,000. As shown in Fig.S2, the larger batch size results in lower inference latency, reaching the minimum at a batch size of 1000. Therefore, a batch size of 1000 was adopted for the reported results. This configuration can reduce time-consuming memory read/write operations and better exploit the GPU’s parallel processing capability.

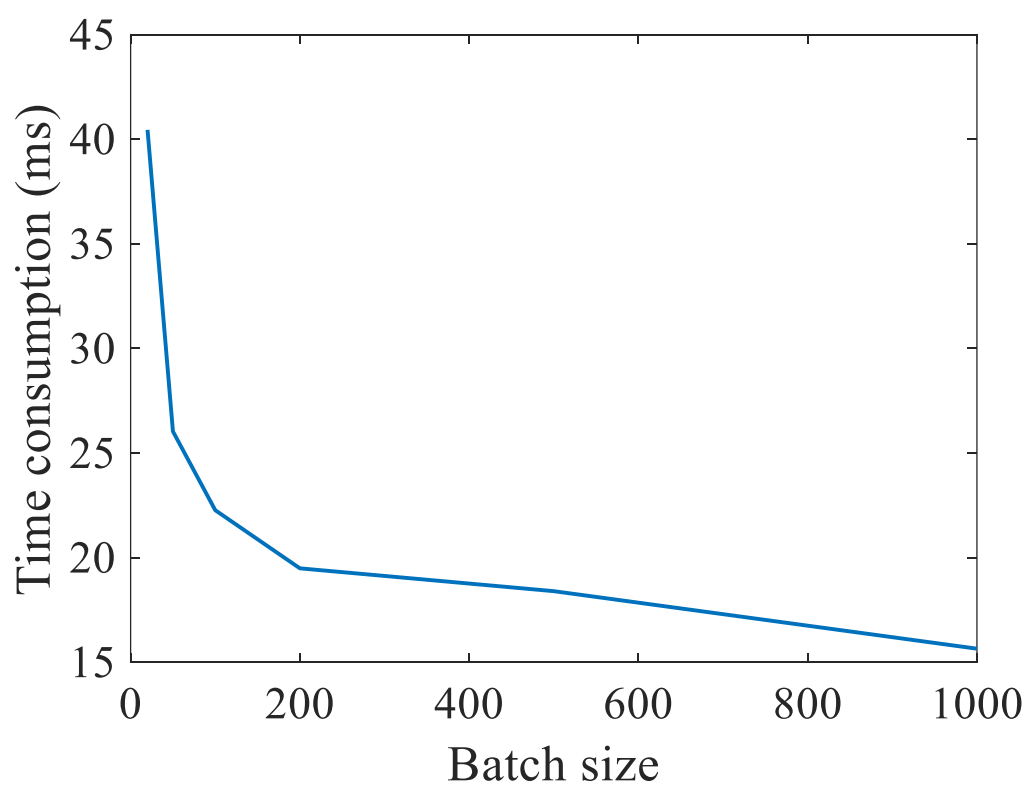

Fig.S2. GPU inference latency versus batch size (total images = 1000).

During the experimental evaluation, a single-image inference was executed beforehand to warm up the GPU and mitigate initialization and kernel compilation

overhead. The inference latency was measured using the high-resolution time.perf_counter() function, with timestamps recorded immediately before invoking the forward pass and after forcing synchronization via .numpy(), thereby guaranteeing completion of all GPU kernels. Since TensorFlow executes GPU operations asynchronously, the explicit .numpy() call ensures that kernel execution, internal scheduling, and device-to-host memory transfers are fully completed before stopping the timer. Therefore, the reported latency includes kernel launch overhead, GPU computation time, synchronization latency, and GPU-to-CPU data transfer, while excluding model loading, graph construction, and data preprocessing stages. Each experiment was repeated five times, and the average execution time was reported. The measured execution time is the mean full-batch inference latency under steady-state conditions. During the benchmark, the GPU operated at an effective core frequency of approximately 2.76 GHz.

In addition, we evaluated the impact of varying the total number of input images on GPU inference performance. As shown in Fig.S3, the total inference latency increases approximately linearly with the number of input images. This trend indicates that the workload scales proportionally with input size. The near-linear growth further confirms that the workload is primarily constrained by memory access rather than peak floating-point throughput, which explains why increasing the total input size results in a proportional increase in execution time.

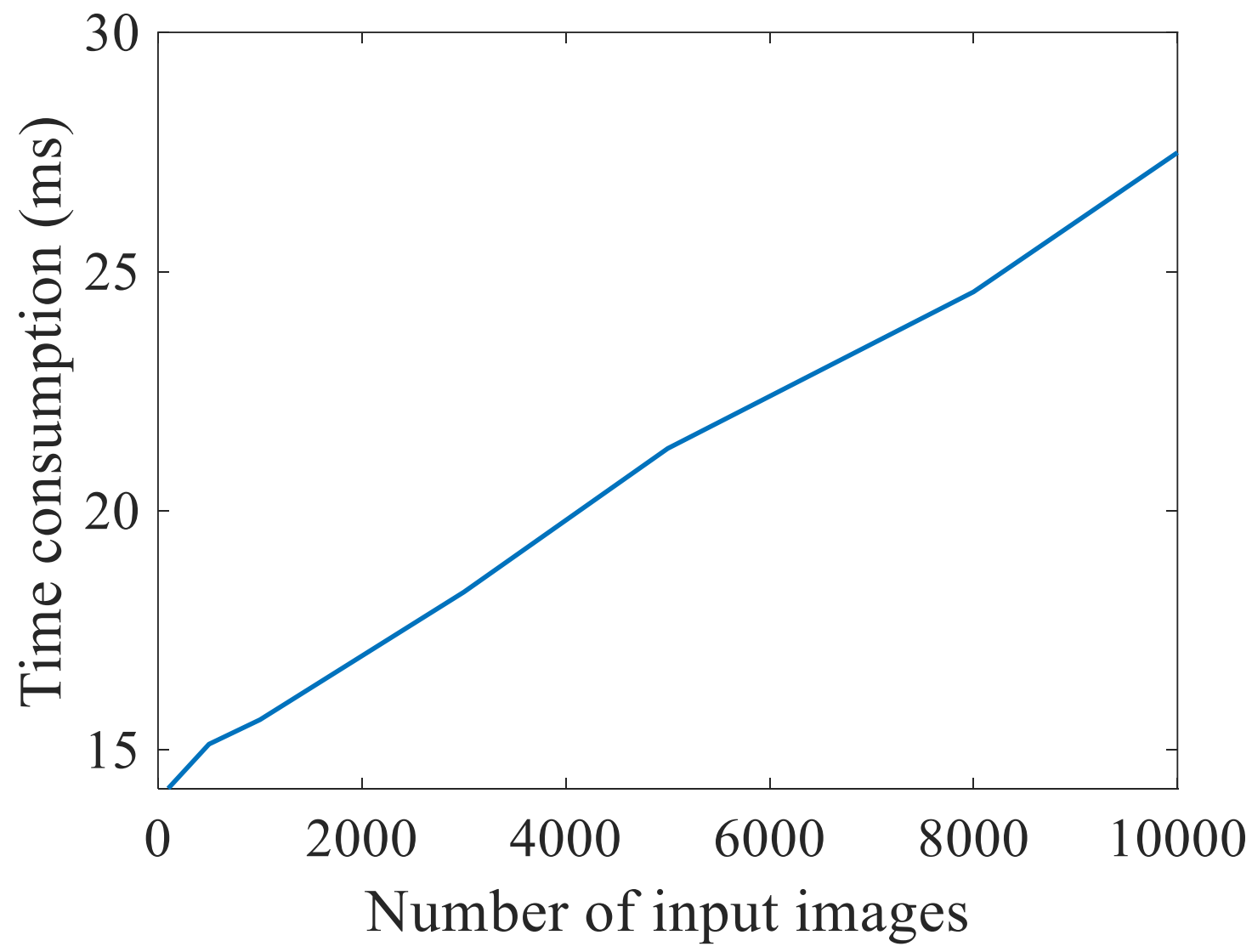

Fig.S3. GPU inference latency versus total number of input images.

Reference：

[1] Poulsen, Mogens Rysholt, et al. "Advances with silica-on-silicon planar waveguides." *Integrated Optics: Devices, Materials, and Technologies VII*. Vol. 4987. SPIE, 2003.

[2] Takahashi, Hiroshi. "Planar lightwave circuit devices for optical communication: present and future." *Active and Passive Optical Components for WDM Communications III*. Vol. 5246. SPIE, 2003.

[3] Dong P, Qian W, Liang H, et al. Thermally tunable silicon racetrack resonators with ultralow tuning power[J]. Optics express, 2010, 18(19): 20298-20304.

[4] Feng X, Wu T, Gao Z, et al. Non-Hermitian hybrid silicon photonic switching[J]. Nature Photonics, 2025, 19(3): 264-270.
